# Recurrence Rates and Risk Factors for Primary Giant Cell Tumors around the Knee: A Multicentre Retrospective Study in China

**DOI:** 10.1038/srep36332

**Published:** 2016-11-09

**Authors:** Pan Hu, Liming Zhao, Huilin Zhang, Xiuchun Yu, Zhen Wang, Zhaoming Ye, Sujia Wu, Shibing Guo, Guochuan Zhang, Jinghua Wang, Xianjia Ning, Yongcheng Hu, Yingze Zhang

**Affiliations:** 1 Department of Orthopaedic Surgery, The Third Hospital of Hebei Medical University, Shijiazhuang, Hebei, China; 2 Department of Oncology of Bone, Tianjin Hospital, Tianjin, China; 3 The Graduate School, Tianjin Medical University, Tianjin, China; 4 Department of Orthopedics, Jinan Military General Hospital, Jinan, China; 5 Department of Orthopedics, Xijing Hospital, Forth Military Medical University, Xi'an, China; 6 Department of Orthopaedics, The Second Affiliated Hospital of Zhejiang University, School of Medicine, Hangzhou, China; 7 Department of Orthopaedics, Nanjing Military General Hospital, Nanjing, China; 8 Department of Orthopaedics, The Second Affiliated Hospital of Inner Mongolia Medical University, Hohhot, China; 9 Department of Oncology of Bone, The Third Hospital of Hebei Medical University, Shijiazhuang, China; 10 Department of Epidemiology, Tianjin Neurological Institute, Tianjin, China

## Abstract

Giant cell tumors of the bone (GCTBs) are commonly diagnosed in Asian populations, usually around the knee. Herein, we aimed to determine the clinical characteristics, local recurrence rates, and relevant risk factors of primary GCTB around the knee. Univariate and multivariate survival analyses were used to identify the risk factors for local recurrence. Four hundred ten patients with primary GCTB around the knee, treated between March 2000 and June 2014, were recruited from 7 institutions in China. The overall local recurrence rate was 23.4%, but was higher in patients aged 20–39 years (28.5%; P = 0.039). The local recurrence rate was the highest in patients treated with intralesional curettage (53.4%), and the lowest in those treated with resection (4.9%). We found a higher risk of tumor recurrence in the proximal fibula compared to the distal femur (hazard ratio: 28.52, 95% confidence interval: 5.88–138.39; P < 0.0001), and in patients treated with curettage compared to those treated with resection (hazard ratio: 12.07, 95% confidence interval: 4.99–29.18; P < 0.0001). Thus, the tumor location must be considered when selecting the optimal surgical treatment approach to reduce the risk of local recurrence and preserve joint function, especially in young patients.

A giant cell tumor of the bone (GCTB) is a primary intramedullary bone tumor composed of mononuclear and giant multinucleated cells that resemble osteoclasts^1^. GCTB is one of the most widely investigated yet perplexing bone tumors. It accounts for 3–8% of primary bone tumors in Western countries; however, it is more common in Asia, where it accounts for approximately 20% of primary bone tumors^2^,^3^,^4^,^5^,^6^,^7^. GCTB is most commonly diagnosed in individuals aged 20–40 years, and >50% of cases occur around the knee^2^,^3^,^8^,^9^,^10^,^11^.

The postoperative recurrence rates of GCTB have been reported to be 10–65%^4^,^5^,^12^,^13^,^14^,^15^,^16^. A previous study from China reported a 12.4% local recurrence rate in patients with primary GCTB located in an extremity^6^. However, this rate was based on data obtained from patients at a single institution over a long time period. On the other hand, large-sample multicentre studies for this type of disease are still lacking, especially for GCTBs occurring at single locations.

Therefore, we conducted a multicentre, nationwide study in China to determine the clinical characteristics, local recurrence rates, and relevant risk factors for primary GCTBs occurring around the knee, and to clarify the appropriate surgical approach for reducing the local recurrence rate and protecting limb function.

## Results

### Demographic and Clinical Characteristics

Table 1 presents the demographic and clinical features of patients with primary GCTB around the knee. Of the included 410 patients, 217 (53%) were men and 193 (47%) were women (male:female ratio, 1.12:1). The mean (standard deviation) age at diagnosis was 35.7 (13.4) years; the majority of patients (57%) were aged between 20 and 39 years. Moreover, GCTB around the knee was more likely to occur in the right-side (53%), and locate distal femur (52%); 48% of the total tumors were categorized as Campanacci grade III tumors. The prevalence of pathological fractures was 34%. In terms of surgery, 24%, 46%, and 30% of patients were treated with intralesional curettage, curettage combined with resection, and en bloc marginal resection, respectively. In terms of the use of adjuvant treatments, polymethylmethacrylate (PMMA), phenol, electrotome, hydrogen peroxide, zinc chloride, and alcohol were used in 24.9%, 17%, 30%, 21%, 15%, and 13% of cases, respectively.

### Local Recurrence Rate According to Clinical Characteristics

As shown in Table 2, the overall local recurrence rate was 23% (26% in men and 21% in women; P = 0.294). Furthermore, the side and location of the tumor, Campanacci grade, presence of pathological fractures, and use of polymethylmethacrylate (PMMA) did not influence the local recurrence rate. However, the rate was significantly higher in patients aged 20–39 years (29%; P = 0.039) and in patients treated with intralesional curettage (53%; P < 0.0001).

### Risk Factors for Local Recurrence

**Figure 1:**
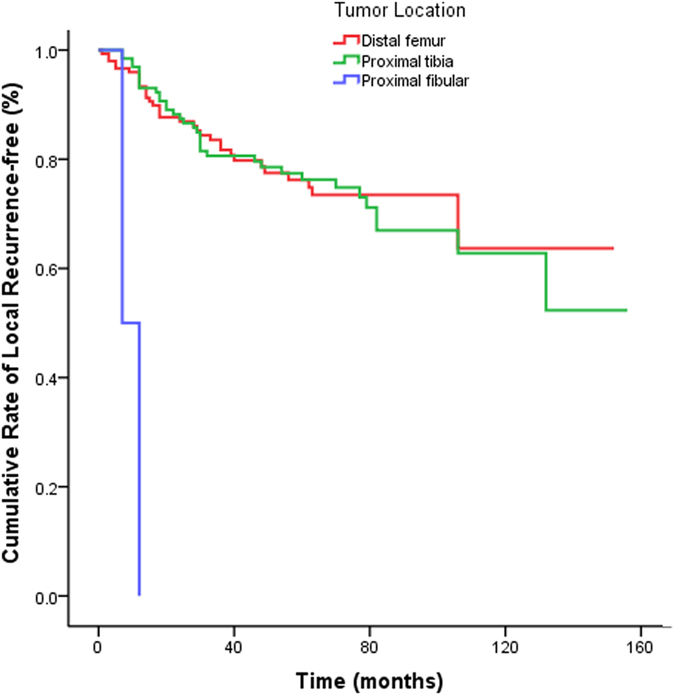
Kaplan-Meier curve of the cumulative rate without local recurrence by tumor location. Reference as tumor located distal femur, the HR (95% CI) of local recurrence was 21.54 (4.89, 94.78) in tumor located in fibular head, P < 0.0001.

**Figure 2:**
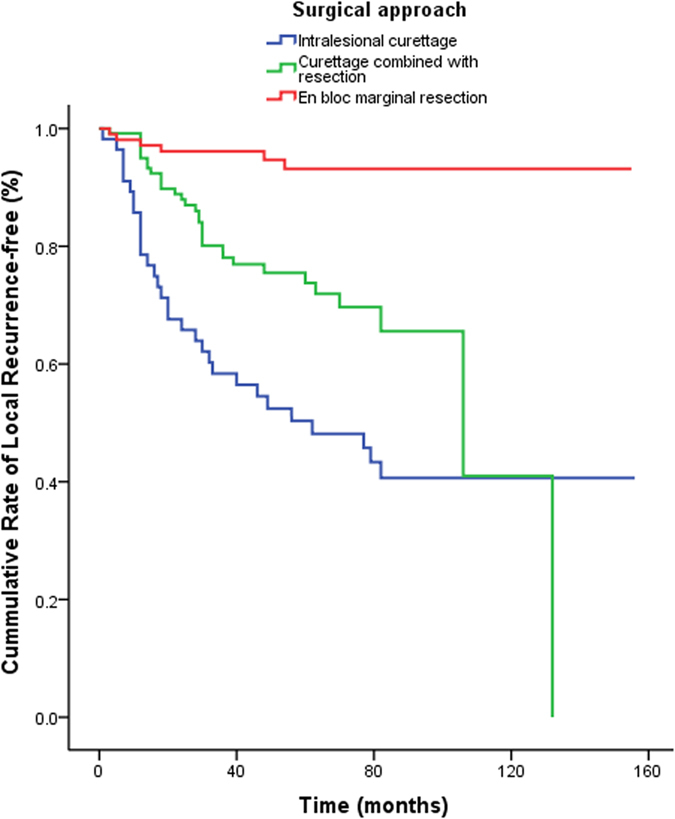
Kaplan-Meier curve of the cumulative rate without local recurrence by surgical approach. Reference as en-bloc resection and marginal resection, there was a HR (95% CI) of 11.25 (4.69, 26.97) in intracystic curettage, and 5.85 (2.45, 13.96) in resection partly with intracystic curettage, all P < 0.0001.

Univariate analysis revealed that local recurrence was significantly associated with tumor location (log rank = 34.599; P < 0.0001) and surgical treatment (log rank = 43.076; P < 0.0001). Tumors located in the proximal fibula had a significantly higher local recurrence rate than those located in the distal femur (hazard ratio [HR]: 21.54, 95% confidence interval [CI]: 4.89–94.78). Moreover, the rate of local recurrence was significantly higher in patients who underwent intralesional curettage (HR: 11.25, 95% CI: 4.69–26.97) or curettage combined with resection (HR: 5.85, 95% CI: 2.45–13.96) compared to those who underwent en bloc marginal resection (all P < 0.0001; Table 3, Figs 1 and 2).

Multivariate analysis indicated that tumor location and surgical treatment were independent risk factors for local recurrence. Tumors located in the proximal fibula had a significantly higher rate of local recurrence compared to those in the distal femur (HR: 28.52, 95% CI: 5.88–138.39). Additionally, treatment with intralesional curettage (HR: 12.07, 95% CI: 4.99–29.18) and curettage combined with resection (HR: 6.39, 95% CI: 2.66–15.36) were associated with significantly higher recurrence rates compared to en bloc marginal resection (all P < 0.0001; Table 3).

### Functional Outcomes

The Musculoskeletal Tumor Society (MSTS) scores were significantly lower in patients with local recurrence than in those without, with median (range) scores of 25.5 (0–30) and 28.0 (1–30), respectively (P = 0.032). The median (range) MSTS scores were 28.0 (13–30), 28.0 (17–30), and 26.0 (0–30) in patients treated with intralesional curettage, curettage combined with resection, and en bloc marginal resection, respectively (P = 0.400).

### Giant Cell Tumor Metastases

Among patients who received at least 12 months of follow-up, 4 developed metastasis (2 cases of pulmonary metastasis, 1 of thoracic vertebra metastasis, and 1 of multifocal bone metastasis). Of these, 3 patients died due to metastatic cancer, including 1 female patient with bilateral pulmonary metastasis, 1 male patient with thoracic vertebra metastasis, and 1 male patient with multifocal bone metastasis. The remaining male patient with unilateral pulmonary metastasis is alive after receiving lesion resection. The pulmonary and overall metastasis rates of GCTB around the knee were 0.7% and 1.3%, respectively.

## Discussion

To the best of our knowledge, this is the first multicentre study to assess the clinical and epidemiological features, local recurrence rates, and factors associated with local recurrence among patients with primary GCTB around the knee by using a representative multicentre GCTB registry in China.

In Western countries, GCTB has been reported to be more likely to occur in women than in men. One previous study reported incidence rates in men and women of 48.5% and 51.5%, respectively^17^, while another reported rates of 44% and 56%, respectively^18^. However, several other studies have reported that GCTB occurs predominately in men, with male:female ratios ranging from 1.27:1 to 1.77:1^6^,^9^,^19^,^20^,^21^. Consistent with the latter studies, we found that GCTB around the knee predominantly occurred in men, with a male:female ratio of 1.12:1. Of note, the male:female ratio in the general population of China was 1.05:1 in 2014^22^, indicating that the higher ratio of male patients with GCTB around the knee is likely not simply reflecting the demographical distribution of the general population. Instead, we postulate that the difference in prevalence according to sex between Asian and Western individuals may be attributed to ethnicity.

GCTB is a highly controversial bone tumor in terms of its pathogenesis, and the reported local recurrence rates range widely, from 12% to 49%^23^,^24^,^25^,^26^,^27^. Previous studies have noted that undergoing surgery was associated with local GCTB recurrence^5^,^25^,^28^,^29^. In particular, curettage has been shown to be strongly associated with an increased risk of local recurrence. In one study, the local recurrence rate in patients with GCTB of the extremities was 2.4-fold higher in those who were treated with curettage (27%) than in those who underwent resection (12%)^28^. In another study, these rates were 25% and 5%, respectively^4^. However, in a series of primary GCTB cases in Canada, the local recurrence rates were 17% overall, 18% in the curettage group, and 16% in the resection group; thus, curettage was not a risk factor for local recurrence in that study^30^. In our study, the local recurrence rates in patients with primary GCTB around the knee were 23% overall, 53% for intralesional curettage, 28% for curettage combined with resection, and 5% for en bloc marginal resection. Compared to en bloc marginal resection, the risk of local recurrence increased by 11.1-fold for intralesional curettage and by 5.4-fold for curettage combined with resection.

Some authors have reported that the recurrence rate varies depending on the tumor location^14^,^15^,^21^. For example, the reported recurrence rate in the distal part of the radius ranges from 20% to 88.9%^18^,^31^,^32^. However, the local recurrence rates around the knee according to location have not been previously reported. In the present study, we found a 27.5-fold increased risk of local recurrence in patients with tumors located in the proximal fibula compared to in the distal femur. The higher rate of local recurrence in the proximal fibula may be explained by its unique anatomical position, as it is surrounded by the peroneal artery and anterior tibial artery and vein; therefore, tumors in the proximal fibula are commonly treated by curettage. On the other hand, it is not possible to remove tumors in the proximal fibula using a high-speed burr, owing to limited bone and thin bone shell. Moreover, as the fibula is not a weight-bearing bone, there is little impact on its functionality after resection, and we therefore recommend that giant cell tumors located in the proximal fibula should be resected.

Previous studies have reported a larger prevalence of Campanacci grade II tumors^33^,^34^,^35^. In contrast, we found higher proportions of both grade II and grade III GCTBs, accounting for 39% and 48% of the cases in the present study, respectively. These data are consistent with those from a previous report from China^6^.

The associations of local recurrence with Campanacci grade and pathological fractures remain uncertain. Several studies have reported that grade III disease is associated with a high recurrence rate^5^,^21^,^36^,^37^,^38^. However, a series from China found a significantly lower recurrence rate for giant cell Campanacci grade III tumors^6^. Similarly, we found that Campanacci grade III tumors exhibited the lowest rate of recurrence (22%), although the difference was not statistically significant. Furthermore, pathological fracture was not associated with local recurrence in this study. This may be explained by the greater proportion of patients with Campanacci grade III tumors (47.5%) who underwent resection compared to those with grade I (9.6%) and grade II tumors (15.0%).In addition, patients with pathological fracture were treated in two stages in this study; first, the fracture was treated through external fixation, and then, the tumor resection was completed. Patients with primary GCTB around the knee did not receive hip replacements in the present study. Thus, a lower local recurrence rate was not associated with tumor severity (grade III) or presence of pathological fracture.

Finally, the prevalence of pathological fractures was 34% in the present study. The pulmonary metastasis rate of GCTB around the knee was 0.7%, while the overall metastasis rate was 1.3%, and these rates were lower than those reported previously^5^,^6^.

PMMA is the most common adjuvant used to fill the tumor cavity, and hypothetically, lowers the risk of local recurrence through its hyperthermic properties^39^. However, in this study, we did not observe a lower local recurrence rate in patients who received PMMA compared to those who did not.

There are some limitations of the present study. First, this was a retrospective multicentre study, and the identification standards for radiological data and clinical staging may have differed among the included institutions. However, this limitation was addressed by using a predefined standardized treatment procedure devised by the Giant Cell Tumor Group of China (GTOC), a committee of experts, and by conducting extensive investigator training at the 7 participating centres. Second, the number of patients was small owing to the low prevalence of GCTB, and all patients were recruited from the 7 centres in China that participated in the study; hence, the included patients might not be representative of all GCTB patients in China.

## Conclusion

To our knowledge, this is the first study based on a large, multicentre GCTB registry system to describe the clinical and epidemiological characteristics and outcomes in GCTB patients in China, and to evaluate the local recurrence rate, including the relevant risk factors, in primary GCTB around the knee. We found that GCTB around the knee occurred more often in men and in young individuals. Simultaneously, we found a higher local recurrence rate in patients aged 20–39 years and in those treated with intralesional curettage. Moreover, the tumor location and type of surgical intervention were independent risk factors for local recurrence; undergoing intralesional curettage and having tumors located in the proximal fibula increased the risk of local recurrence in patients with primary GCTB around the knee. The anatomical position of the proximal fibula may contribute to this increased risk of local recurrence, as it is surrounded by the peroneal artery and anterior tibial artery and vein. Thus, it is crucial to select the appropriate surgical treatment approach by considering the tumor location in order to reduce the risk of local recurrence and preserve knee function, especially for young patients with high-risk tumor locations.

## Methods

### Patient Selection

We recruited patients with primary GCTB around the knee, including the distal femur, proximal tibia, proximal fibular, and patella, from the GTOC between March 2000 and February 2015. The GTOC is an association of physicians from orthopaedic oncology centres located in different regions of China, who treat giant cell tumors. Seven centres established the GTOC in 2005, with 5 more joining in 2016; the data from these centres were thus not included in the present study. The aim of this group is to standardize the diagnosis and treatment of giant cell tumors in China. The current standardized procedure was devised by GTOC experts by taking into account the presence of pathological fracture, shifting of the articular surface, the Campanacci grade, the growth level of the tumor (whether the tumor has grown with or without breaking through the articular surface), and tumor volume.

We reviewed the patients’ medical records retrospectively. All the patients with a confirmed diagnosis of GCTB were included in this study. As a result, we extracted 510 patients with a confirmed histological diagnosis of benign GCTB. Of these, 410 (80.4%) patients with primary GCTB around the knee were recruited for this study, while 100 (19.6%) with recurrent tumors and who were treated elsewhere were excluded. Moreover, we excluded patients who were suspected of having GCTB preoperatively but whose diagnoses were not confirmed postoperatively, and cases of recurrent GCTB that were treated non-surgically were also not analysed in this study.

In the present study, we excluded pregnant women with GCTB owing to poor outcomes. There were 8 pregnant patients diagnosed with GCTB. Of these, 5 patients had tumors in the distal femur, and 3 had tumors in the proximal femur. Generally, the recurrence of GCTB occurred within 2 years after resection. However, in this study, local recurrence occurred after 8 years in 1 pregnant patient; the cause was unclear.

The clinical and imaging data of primary GCTBs around the knee were reviewed retrospectively.

The following information was recorded for all patients: tumor side (left or right), tumor location (distal femur, proximal tibia, proximal fibula, and patella), Campanacci stage (grade I, II, or III), pathological fracture (yes or no), surgical treatment method (intralesional curettage, curettage combined with resection, or en bloc marginal resection), and application of PMMA cement.

All investigative protocols were approved by the ethics committee of Tianjin Hospital. The procedures were performed according to approved guidelines, and written informed consent was obtained from each patient.

### Surgical Treatment

The surgical techniques were based on the severity of the tumor and included intralesional curettage, curettage combined with resection, and en bloc marginal resection^20^. One or more adjuvants, including PMMA, phenol, electrotome, hydrogen peroxide, zinc chloride, and alcohol, were used during the surgical procedures.

**Figure 3:**
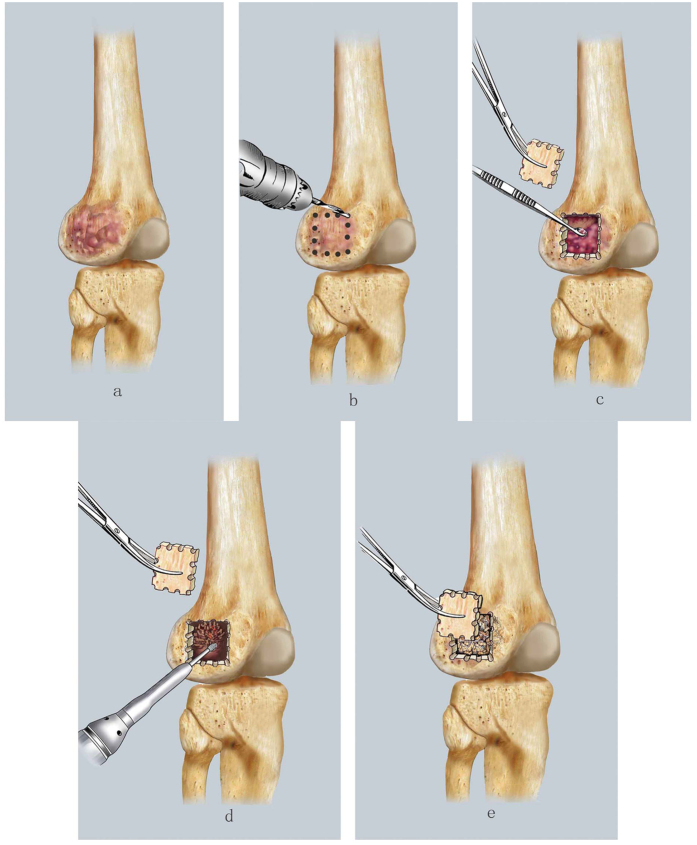
The diagrammatic drawing of intracystic curettage: (**a**) Indications: with a localized lesion, no broken or mild broken the cortical bone, without obvious soft tissue mass. (**b**) A window in the cortical bone was made. (**c**) To remove mass using a series of curettes of various sizes. (**d**) To polish the residual tumor cavity with a high-speed burring until reaching the normal cortical bone. (**e**) To fill the residual tumor cavity with allogenic particle bone graft and covered the windowed cortical bone.

Intralesional curettage was indicated for patients with a localized lesion,that is, lesions without a comminuted pathological fracture in the backbone, without an intra-articular fracture following obvious shifting of the articular surface, without a tumor breaking through the articular surface, classified as Campanacci grade I/II, and with the appropriate tumor volume. With this procedure, a window in the cortical bone is created and the mass is resected by using a series of curettes of various sizes. The residual tumor cavity is then polished with a high-speed burr until reaching the normal cortical bone. Subsequently, allogeneic particle bone is grafted to fill the residual tumor cavity, and the excised section that created the original cortical bone window is finally reattached (Fig. 3).

Moreover, PMMA cement and a steel plate were used to fill tumor cavities, especially for cases with large tumor volumes.

**Figure 4:**
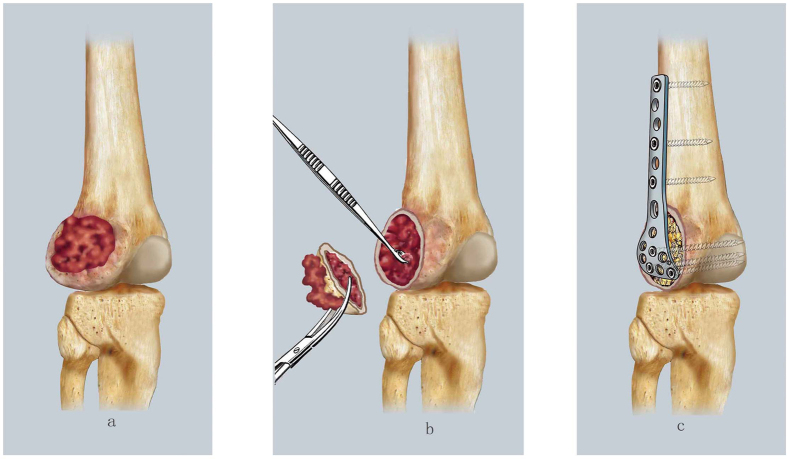
The diagrammatic drawing of curettage combined with resection: (**a**) Indications: with an extensive lesion, with around soft tissue mass, the part broken cortical bone without possible of reserve, with a tumor involved the articular cavity or cruciate ligament. (**b**) To remove the cortical bone and soft tissue mass without possible of reserve, and continued to dispose the tumor cavity using curette and a high-speed burr. (**c**) To fill the cavitary bone defects with allogenic particle bone graft, and internal fixation using an anatomical bone plate.

Curettage combined with resection was performed in patients with extensive lesions, including lesions classified as Campanacci grade III, with a large tumor volume or focus, a tumor breaking through the articular surface, and involving the articular cavity. In this procedure, the cortical bone and soft tissue mass are removed, and the tumor cavity is excavated using a curette and high-speed burr. Subsequently, cavitary bone defects are filled with allogeneic particle bone grafts, and an anatomical bone plate is used for internal fixation (Fig. 4).

**Figure 5:**
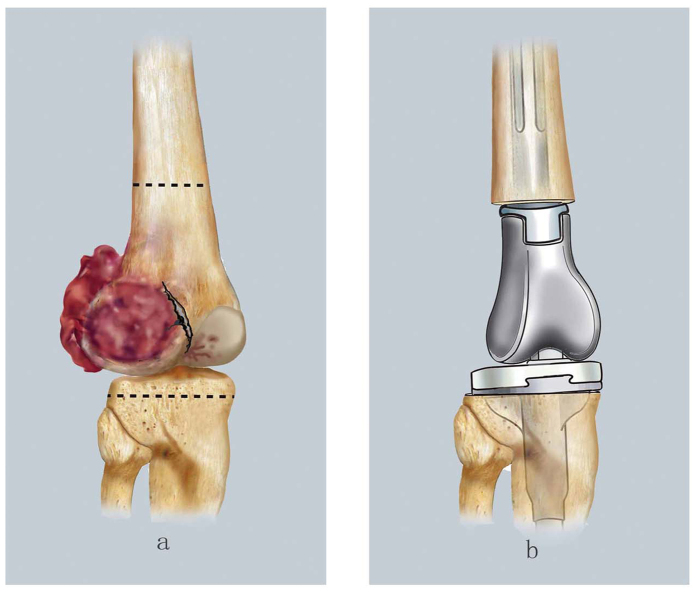
The diagrammatic drawing of en bloc marginal resection: (**a**) Indications: with extensive bone cortex lesions together with around large soft tissue mass. An osteotomy plane was confirmed based on preoperative magnetic resonance imaging (dashed line indicated the osteotomy plane). (**b**) To resect the en bloc tumor, and to reconstruct the knee using an articulated prosthesis.

En bloc marginal resection was indicated for patients with extensive bone cortex lesions along with large soft tissue masses. With this procedure, the osteotomy plane is confirmed via preoperative magnetic resonance imaging and the tumor is resected en bloc. An articulated prosthesis is used to reconstruct the knee (Fig. 5).

### Follow-up and Functional Outcomes

Patients were followed-up every 3 months for the first 2 years post-surgery, every 6 months for the following 3 years, and finally every 12 months in the subsequent 5 years. Telephone interviews were available only after 5 years of follow-up. The MSTS score (total possible score = 30) was used to assess functional outcomes^40^.

**Figure 6:**
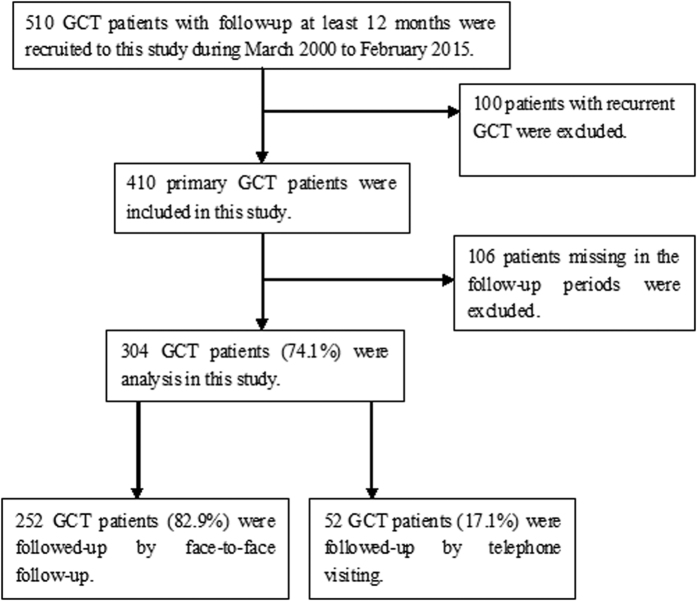
Flow chat of patients’ selection. Of the 410 included patients, 304 completed ≥12 months of follow-up (response rate, 74.1%), with a median follow-up time of 55 months (range, 12–188 months). Of these patients, 252 (82.9%) received face-to-face follow-up with physical and radiological examinations at the 7 participating hospitals; 52 (17.1%) were also contacted via telephone, with their physical and radiological examinations performed at local hospitals.

Of the 410 included patients, 304 completed ≥12 months of follow-up (response rate, 74.1%), with a median follow-up time of 55 months (range, 12–188 months). Of these patients, 252 (82.9%) received face-to-face follow-up with physical and radiological examinations at the 7 participating hospitals; 52 (17.1%) were also contacted via telephone, with their physical and radiological examinations performed at local hospitals (Fig. 6).

### Statistical Methods

Clinical features were assessed, including the tumor side and location, Campanacci grade, presence of pathological fracture, and treatment technique. The local recurrence rates and relevant risk factors were analysed according to the clinical characteristics in patients with at least 12 months of follow-up. Continuous variables are summarized as means (standard deviations) or medians (ranges), and differences between the groups were assessed using the Student t-test or Mann-Whitney U test, as appropriate. Categorical variables are presented as case numbers (percentages), and the chi-square test was used to assess the differences in clinical characteristics and surgical treatment according to local recurrence. Kaplan-Meier survival estimates were used for univariate survival analyses, while the Cox proportional hazards regression model was used for multivariate analysis of risk factors for local recurrence that were found to be significant in the univariate analysis. Risk factors for local recurrence are presented using HRs with 95% CIs. All statistical analyses were performed using SPSS version 15.0 (SPSS Inc., Chicago, IL), and two-tailed P values <0.05 were considered statistically significant.

## Additional Information

**How to cite this article**: Hu, P. *et al.* Recurrence Rates and Risk Factors for Primary Giant Cell Tumors around the Knee: A Multicentre Retrospective Study in China. *Sci. Rep.* **6**, 36332; doi: 10.1038/srep36332 (2016).

**Publisher’s note**: Springer Nature remains neutral with regard to jurisdictional claims in published maps and institutional affiliations.

## Figures and Tables

**Table 1:** The demographical and clinical characteristics in patients with primary GCTB around the knee.

Categories	Number	Percentage
Total, n (%)	410	—
Men	217	52.9
Women	193	47.1
Age, years, mean (SD)	35.7 (13.4)	—
Age group, n (%)		
<20 years	39	9.5
20–39 years	235	57.3
≥ 40 years	136	33.2
Side, n (%)		
Left Knee	191	46.6
Right Knee	219	53.4
Location, n (%)		
Distal femur	213	52.0
Proximal tibia	183	44.6
Proximal fibular	11	2.7
Patella	3	0.7
Campanacci grade, n (%)		
I	52	12.7
II	160	39.0
III	198	48.3
Pathologic fracture, n (%)		
No	271	66.1
Yes	139	33.9
Surgical approach, n (%)		
Intralesional curettage	98	23.9
Curettage combined with resection	189	46.1
En bloc marginal resection	123	30.0
PMMA, n (%)		
Yes	102	24.9
No	308	75.1

PMMA indicated polymethylmethacrylate.

**Table 2:** The demographical and clinical characteristics in patients with primary GCTB around the knee by local recurrence.

Categories	Recurrence	No-Recurrence	P
Total, n (%)	71 (23.4)	233 (76.6)	—
Age, years, mean (SD)	32.68 (12.04)	36.36 (13.65)	0.042
Gender, n (%)			0.294
Men	41 (25.8)	118 (74.2)	
Women	30 (20.7)	115 (79.3)	
Age group, n (%)			0.039
<20 years	4 (14.3)	24 (85.7)	
20–39 years	51 (28.5)	128 (71.5)	
≥40 years	16 (16.5)	81 (83.5)	
Side, n (%)			0.378
Left Knee	35 (25.7)	101 (74.3)	
Right Knee	36 (21.4)	132 (78.6)	
Location, n (%)			0.609
Distal femur	34 (21.8)	122 (78.2)	
Proximal tibia	35 (26.1)	99 (73.9)	
Proximal fibular	2 (18.2)	9 (81.8)	
Patella	0	3 (100.0)	
Campanacci grade, n (%)			0.475
I	8 (33.3)	16 (66.7)	
II	25 (23.1)	83 (76.9)	
III	38 (22.1)	134 (77.9)	
Pathologic fracture, n (%)			0.217
No	44 (26.0)	125 (74.0)	
Yes	27 (20.2)	108 (80.0)	
Surgical approach, n (%)			<0.0001
Intralesional curettage	31 (53.4)	27 (46.6)	
Curettage combined with resection	34 (27.6)	89 (72.4)	
En bloc marginal resection	6 (4.9)	117 (95.1)	
PMMA, n (%)			0.516
Yes	18 (40.9)	26 (59.1)	
No	45 (35.4)	82 (64.6)	

PMMA indicated polymethylmethacrylate.

**Table 3:** The adjusted hazards ratio of risk factors of local recurrence in patients with primary GCTB around the knee.

Risk factors	Reference	Univariate Analysis		Multivariate Analysis	
		Unadjusted HR (95% CI)	P	Adjusted HR (95% CI)	P
Men	Women	0.82 (0.51, 1.31)	0.404		
Age	—	0.98 (0.96, 1.00)	0.068		
Left Knee	Right Knee	0.79 (0.50, 1.26)	0.329		
Location	Distal femur				
Proximal tibia		1.06 (0.66, 1.70)	0.817	0.81 (0.50, 1.32)	0.400
Proximal fibular		21.54 (4.89, 94.78)	<0.0001	28.52 (5.88, 138.39)	<0.0001
Campanacci grade	I				
II		0.59 (0.26, 1.30)	0.187		
III		0.55 (0.26, 1.19)	0.130		
Pathologic fracture	No	0.66 (0.41, 1.07)	0.092		
Surgical approach	En bloc marginal resection				
Intralesional curettage		11.25 (4.69, 26.97)	<0.0001	12.07 (4.99, 29.18)	<0.0001
Curettage combined with resection		5.85 (2.45, 13.96)	<0.0001	6.39 (2.66, 15.36)	<0.0001
PMMA	No	1.16 (0.75, 1.77)	0.516		

PMMA indicated polymethylmethacrylate.
